# Supplementary material for: Plastics and sustainable purchase decisions in a circular economy: The case of Dutch food industry
Source: PLoS One. 2020 Sep 28;15(9):e0239949. doi: 10.1371/journal.pone.0239949 (PMC7521728; doi:10.1371/journal.pone.0239949)
Supplement: S1 Questionnaire — (PDF) [file pone.0239949.s001.pdf]

The Circular Economy of plastic food packaging (CONSUMERS)

Dear respondent,

This online survey is about the transition towards a Circular Economy of plastic food packaging. It will take approximately 5 minutes to complete. You are free to stop participating at any point during this survey. The survey is being conducted as part of the Master's Degree in Economics at the Universidad de Granada (Spain).

1. **Please, enter your name**
3. [ ]
4. **Please, enter your e-mail address**
5. [ ]
6. **Are you a male or female? ***
    a. Female
    b. Male
    c. Prefer not to say
7. Anders:
8. [ ]

PART B

1. **How old are you? ***
    1.1. Under 18
    1.2. 18-24
    1.3. 25-34
    1.4. 35-44
    1.5. 45-54
    1.6. 55-64
    1.7. 65-74
    1.8. 75-84
    1.9. 85 or older

2. **What describes your current living situation best? ***
    2.1. I am Dutch and live in the Netherlands
    2.2. I am Dutch but I do not live in the Netherlands
    2.3. I am not Dutch but I do live in the Netherlands
    2.4. I am not Dutch and I do not live in the Netherlands

3. **What is your yearly income before tax? ***
    3.1. Between €0 and €10.000
    3.2. Between €10.000 and €20.000
    3.3. Between €20.000 and €30.000
    3.4. Between €30.000 and €40.000
    3.5. €40.000 or higher

4. **What is the highest level of education you have completed?**

4.1. Primary education
4.2. Secondary education
4.3. Intermediate vocation training (MBO)
4.4. Higher vocation training (HBO)
4.5. Bachelor
4.6. Master PhD
4.7. Other

Environmental issues

You will now be asked to fill out a couple of questions about environmental issues

5. How important are environmental/sustainability considerations to you, personally? *

Not at all important 1 2 3 4 5 6 7 Extremely important

6. To what extent are you familiar with the following terms?

1. Sustainability
2. Circular Economy
3. Cradle-to-cradle
4. Closed-loop-recycling
5. Disposable plastics
6. Micro-plastics
7. Primary recycling
8. Waste hierarchy (Ladder van Lansink)
9. Extended Producer Responsibility
10. Corporate Social Responsibility
11. Life Cycle Assessment
12. Sustainability
13. Circular Economy
14. Cradle-to-cradle
15. Closed-loop-recycling
16. Disposable plastics
17. Micro-plastics
18. Primary recycling
19. Waste hierarchy (Ladder van Lansink)
20. Extended Producer Responsibility
21. Corporate Social Responsibility
22. Life Cycle Assessment

7. How important are the following factors for you, when buying FOOD (packed in plastic packaging) in the supermarket?

1. Price
2. Quality
3. Branding
4. Environmentally-friendly production
5. Recyclabiltiy at end of use

6. Repairabiltiy
7. Durabiltiy
8. Upgradeabilty
9. Price
10. Quality
11. Branding
12. Environmentally-friendly production
13. Recyclabiltiy at end of use
14. Repairabiltiy
15. Durabiltiy
16. Upgradeabilty

PART C

8. Please indicate how serious you think each of the following environmental issues are (1=not serious, 7=extremely serious) *

Not at all important 1 2 3 4 5 6 7 Extremely important

1. Plastic in the ocean
2. The amount of plastic waste produced
3. The amount of general waste going to landfill
4. Water pollution
5. Endangered species and biodiversity
6. Natural resources depletion (forest, water, energy)
7. Air pollution
8. Water shortages
9. Climate change (global warming)
10. Plastic in the ocean
11. The amount of plastic waste produced
12. The amount of general waste going to landfill
13. Water pollution
14. Endangered species and biodiversity
15. Natural resources depletion (forest, water, energy)
16. Air pollution
17. Water shortages
18. Climate change (global warming)
19. Plastics

You will now be asked to fill out a couple of questions about plastic

9. Do you care about plastic pollution?

Yes
No
Have not thought about it

10. Please record the first two NEGATIVE words that come to mind when you hear the word 'plastic' *

11.

12. **Please record the first two POSITIVE words that come to mind when you hear the word 'plastic' ***
13. [ ]

14. **Do you reduce your use of 'on-to-go' plastic (e.g. bring your own take-away coffee cup, bring your own take-away food container)**
    1. Never
    2. Rarely
    3. Roughly 30% of the time
    4. Roughly 50% of the time
    5. Always

15. **Do you reduce your use of plastic packaging (e.g. buy at a 'packaging-free, zero-waste' store; avoid packaged personal care products)**
    1. Never
    2. Rarely
    3. Roughly 30% of the time
    4. Roughly 50% of the time
    5. Always

16. **Do you reduce your use of non-disposable plastic packaging (e.g. replace plastic containers with glass, buy wooden householdgoods as opposed to plastic goods)**
    1. Never
    2. Rarely
    3. Roughly 30% of the time
    4. Roughly 50% of the time
    5. Always

Part D.
Plastic food packaging

You will now be asked to fill out a couple of questions about plastic food packaging
Please rate plastic food packaging against the following traits: inconvenient/convenient *

Highly Inconvenient Highly Convenient
1 2 3 4 5 6 7

17. **Please rate plastic food packaging against the following traits: non useful/useful ***
    Non useful Useful
    1 2 3 4 5 6 7

18. **Please rate plastic food packaging against the following traits: bad/good ***
    Bad Good
    1 2 3 4 5 6 7

19. **Please rate plastic food packaging against the following traits: harmful/beneficial**

    Harmful Beneficial
    1 2 3 4 5 6 7

Recycling

20. You will now be asked to fill out a couple of questions about recycling of plastic food packaging

21. What percentage of globally used plastic packaging is recycled in 2016?

____

22. To what extent do you separate your plastic waste?

Never — Always

Never						Always
1	2	3	4	5	6	7

23. What percentage of the population in the Netherlands do you think separated their plastic waste for recycling in 2018?

____

24. I believe everyone in the Netherlands should be separate plastic waste for recycling

Strongly disagree						Strongly agree
1	2	3	4	5	6	7
