# Supplementary material for: Plastics and sustainable purchase decisions in a circular economy: The case of Dutch food industry
Source: PLoS One. 2020 Sep 28;15(9):e0239949. doi: 10.1371/journal.pone.0239949 (PMC7521728; doi:10.1371/journal.pone.0239949)
Supplement: Attachment — Submitted filename: response to reviewer 1407.docx [file pone.0239949.s003.docx]

COMMENTS TO REVIEWERS

Reviewer 1.

1. Section 1 and 2 are very lengthy. I would suggest to merge these sections under a single heading of Introduction and limit it to two to three pages.

In order that the text not to be so extensive, the introductory chapter was modified, removing content, reducing it to a page and a half. The second and third sections have been merged, also cutting their length.

2. Latest references should be incorporated.

The references of the work have been updated, including the following:

• Blomsma, F. and G. Brennan (2017). The emergence of circular economy: a new framing around prolonging resource productivity. Journal of Industrial Ecology, 21 (3), pp. 603-614.

• Mugge, R. (2018), Product Design and Consumer Behavior in a Circular Economy, Sustainabilty, 2018, 10 (10, pp. 1-4.

• Nuñez-Cacho, P. Górecki, J. Molina-Moreno, V and F.A. Corpas-Iglesias (2018), What Gets Measured, Gets Done: Development of a Circular Economy Measurement Scale for Building Industry. Sustainability 2018, 10, 2340.

• Prieto-Sandoval, C. Jaca and M. Ormazabal (2018), Towards a consensus on the circular economy. Journal of Cleaner Production, 179. 605-615.

3. Plastic production data of 2015 is mentioned. It is suggested to provide data of 2019.

The data on world plastic production for 2015 has been updated to 2018.

REVIEWER 2

1. There are two “The” in the title of the manuscript as highlighted above, which seems to be inappropriate. Authors are advised to keep only one.

The title has been revised with a translator, including now just one “the”. So, the final title is “Plastics and Sustainable Purchase Decisions in a Circular Economy: The Case of Dutch Food Industry”

2. Initial lines of the abstract are well written but there are no statements of findings. The results of work should be written in the abstract to make it more prominent.

Several new lines have been added to the abstract explaining the results obtained.

3. All the keywords are in upper case. The authors are advised to write them in lower case.

The keywords has been modified according the suggestion of the reviewer.

4. The list of abbreviations or symbols used should be placed either at beginning or at the end of manuscript.

The list of abbreviations has been added at the end of the paper.

5. Legend of figure-1 should contain “that”. It should be like “Factors influencing sustainable purchase decisions”

Legend of figure 1 has been modified to “Figure 1: Factors influencing sustainable purchase decisions”

6. The sections like “practical implications” and Limitations and future prospective” presented in page-18 are not well organized. The authors are advised to reorganize and present the sections before the conclusion.

The section on practical implications and limitations has been reorganized, placing them before the conclusion.

7. Most of the references are found to be very old. Authors should refer and cite some latest papers related to their research. Minor revisions given above must be incorporated in the manuscript for consideration.

We´ve added new references of the last years:

• Blomsma, F. and G. Brennan (2017). The emergence of circular economy: a new framing around prolonging resource productivity. Journal of Industrial Ecology, 21 (3), pp. 603-614.

• Mugge, R. (2018), Product Design and Consumer Behavior in a Circular Economy, Sustainabilty, 2018, 10 (10, pp. 1-4.

• Nuñez-Cacho, P. Górecki, J. Molina-Moreno, V and F.A. Corpas-Iglesias (2018), What Gets Measured, Gets Done: Development of a Circular Economy Measurement Scale for Building Industry. Sustainability 2018, 10, 2340.

• Prieto-Sandoval, C. Jaca and M. Ormazabal (2018), Towards a consensus on the circular economy. Journal of Cleaner Production, 179. 605-615.
