# Supplementary material for: Plastics and sustainable purchase decisions in a circular economy: The case of Dutch food industry
Source: PLoS One. 2020 Sep 28;15(9):e0239949. doi: 10.1371/journal.pone.0239949 (PMC7521728; doi:10.1371/journal.pone.0239949)
Supplement: Attachment — Submitted filename: Reviewer comments.docx [file pone.0239949.s002.docx]

**Plos one Journal**

**Manuscript Title:	Plastics and Sustainable Purchase Decisions in a Circular Economy:	The Case of the Holland Food Industry**

**Manuscript No:	PONE-D-20-10526**

**REVIEWER’S COMMENTS**

This is an interesting study that provides fundamental approach for Plastics and Sustainable Purchase Decisions in a Circular Economy. However, minor revisions are suggested for improvement of the manuscript according to the following comment lines.

1. There are two “The” in the title of the manuscript as highlighted above, which seems to be inappropriate. Authors are advised to keep only one.
2. Initial lines of the abstract are well written but there are no statements of findings. The results of work should be written in the abstract to make it more prominent.
3. All the keywords are in upper case. The authors are advised to write them in lower case.
4. The list of abbreviations or symbols used should be placed either at beginning or at the end of manuscript.
5. Legend of figure-1 should contain “that”. It should be like “Factors influencing sustainable purchase decisions”
6. The sections like “practical implications” and Limitations and future prospective” presented in page-18 are not well organized. The authors are advised to reorganize and present the sections before the conclusion.
7. Most of the references are found to be very old. Authors should refer and cite some latest papers related to their research.

**Recommendation:**	The overall work is nicely presented in this manuscript and the claim is significant. So it should be considered for publication with the minor revisions.

Best regards,

**Shankar Swarup Das**

**Reviewer**
